# Perturbing H-NS function reveals roles in restricting virulence heterogeneity and pathogen adaptation

**DOI:** 10.1371/journal.ppat.1014488

**Published:** 2026-08-10

**Authors:** Leah McLelland, Madison Spratt, Dakshya Karki, Anika Marand, Miracle Burt, Jonathan Zhao, Keara Lane

**Affiliations:** 1 Department of Molecular Biosciences, Northwestern University, Evanston, Illinois, United States of America; 2 Interdisciplinary Biological Sciences Graduate Program, Northwestern University, Evanston, Illinois, United States of America; 3 National Institute for Theory and Mathematics in Biology, Northwestern University and The University of Chicago, Chicago, Illinois, United States of America; University of Vermont, UNITED STATES OF AMERICA

## Abstract

Xenogeneic silencers, such as histone-like nucleoid structuring protein (H-NS), are critical for maintaining horizontally acquired genes in bacterial genomes and minimizing fitness costs associated with inappropriate expression. For bacterial pathogens, this has enabled the acquisition of costly virulence regulons, with H-NS balancing the need for tight silencing with rapid expression in host environments. For *Salmonella enterica* serovar Typhimurium (STm), survival in these environments relies on phenotypic heterogeneity in virulence gene expression and evolutionary adaptation. Although complete loss of *hns* is highly deleterious in STm, how subtle impairments to this global silencer disrupt heterogeneity in virulence gene expression and alter adaptation to host environments remains poorly understood. Here, we identify an STm *hns* hypomorph strain and find that its reduced H-NS DNA-binding affinity increases the proportion of virulence-expressing cells, resulting in enhanced epithelial cell infection *in vitro*. Furthermore, through experimental evolution in intracellular-like conditions *in vitro*, we demonstrate that both wild-type and mutant populations converge on disrupting the SPI-2 virulence regulon to improve fitness; however, the mutant population also acquires distinct adaptive mutations to resolve the underlying dysregulation in gene expression. These results suggest that H-NS sets single-cell virulence activation thresholds and that even minor disruptions to its silencing function impact pathogen adaptation, highlighting its role as a critical evolutionary buffer.

## Author summary

Bacteria can acquire new genes from their environment in a process called horizontal gene transfer. While this enables bacteria to acquire new functions, including virulence genes required to cause disease, expression of these new genes must be tightly controlled to avoid fitness costs. Bacteria use proteins called xenogeneic silencers to keep these genes switched off until they are needed. In the pathogen *Salmonella*, the xenogeneic silencer H-NS is critical for controlling the expression of horizontally acquired genes. Strains with complete loss of H-NS have severe growth defects and widespread misregulation of horizontally acquired genes. Here, we identified a strain of *Salmonella* carrying a mutation in H-NS that reduces its ability to bind DNA. Using this strain, we show that impaired H-NS function is sufficient to increase the fraction of cells expressing virulence genes and to enhance bacterial invasion of epithelial cells in culture. We also show that when bacteria with this impaired H-NS have to adapt to intracellular-like conditions, optimizing growth requires both resolving the gene expression dysregulation caused by impaired H-NS silencing and reducing virulence gene expression. Together, these findings reveal that even minor impairments to H-NS function have widespread consequences, altering virulence activation thresholds and pathogen adaptation.

## Introduction

Xenogeneic silencers are essential regulators of horizontally acquired genes in bacteria, enabling their maintenance in the genome while avoiding fitness costs associated with inappropriate expression [1–3]. In bacterial pathogens, horizontally acquired genes frequently encode virulence factors such as type III secretion systems (T3SS) or toxins. Thus, maintaining the appropriate level of silencing is particularly critical, as insufficient silencing imposes fitness costs that can lead to virulence gene loss, while excessive silencing prevents the rapid expression needed to adapt and survive in host environments. Here, we examine how perturbing one such xenogeneic silencer, histone-like nucleoid structuring protein (H-NS), affects virulence gene expression heterogeneity and evolutionary adaptation in the intracellular bacterial pathogen *Salmonella enterica* serovar Typhimurium (STm).

Among xenogeneic silencers, H-NS proteins are found in a subset of γ-proteobacteria and have been extensively studied in *E. coli* and STm [3–5]. H-NS is an abundant nucleoid-associated protein composed of an N-terminal oligomerization domain and a C-terminal DNA-binding domain (DBD) connected via a flexible linker. The DBD preferentially binds to AT-rich DNA sequences characteristic of horizontally acquired genes, including virulence genes [6,7]. Binding is mediated by the highly conserved Q/RGR motif in the DBD, which acts as an AT-hook, inserting into the minor groove to form direct contacts with DNA [8]. Upon binding to high-affinity nucleation sites, H-NS forms higher-order complexes via its oligomerization domain [9]. The resulting H-NS nucleoprotein filaments can compact and bridge large regions of DNA, thereby silencing gene expression by occluding or trapping RNA polymerase [4,10–12]. Numerous countersilencing mechanisms exist to relieve this silencing, including displacement of H-NS by countersilencing proteins, blockade of H-NS polymerization, changes in DNA topology, transcription-induced supercoiling, including that arising from spurious transcription, and changes in H-NS concentration [13–18].

H-NS-mediated silencing of horizontally acquired genes is essential for STm fitness. Loss of *hns* causes widespread misregulation of gene expression and significant growth defects, with ∆*hns* strains viable only in combination with suppressor mutations in genes such as *rpoS* (the stationary-phase sigma factor), *phoP* (a two-component system response regulator), or deletions of the SPI-1 virulence regulon [6,7,19,20]. Several virulence regulons are misregulated in ∆*hns* strains; deletion of the virulence regulator *ssrA* partially rescues the growth defect of the Δ*hns* strain, demonstrating that H-NS-mediated silencing of virulence genes is critical for STm fitness [7]. However, for STm to survive in specific host environments, this silencing must be rapidly relieved. Countersilencing proteins, which include standalone DNA-binding proteins as well as response regulators, can displace H-NS to enable virulence gene expression in response to specific host signals [13–15].

Virulence gene expression is highly heterogeneous in STm, even under uniform inducing conditions. This non-genetic variation, referred to as phenotypic heterogeneity, arises primarily from stochastic gene expression [21,22]. Generating distinct subpopulations of virulence-expressing and non-expressing bacteria enables bet-hedging or division-of-labor strategies that improve survival in host environments [23,24]. In STm, phenotypic heterogeneity is associated with two major virulence regulons encoded in *Salmonella* Pathogenicity Islands 1 and 2 (SPI-1 and SPI-2) [25]. Expression of SPI-1, which is required for invasion of the intestinal epithelium, is bistable and incurs a fitness cost [26–29]. Both SPI-1(+) and SPI-1(-) subpopulations are required for a productive infection *in vivo*, and perturbing this ratio impairs infection outcomes. Expression of SPI-2, which is required for survival and replication within host cells, is bimodal and probabilistically tuned to the strength of intracellular-like signals, including pH and magnesium concentration, *in vitro* [28,30–35]. While the significance of phenotypic heterogeneity in STm virulence gene expression is well established, the extent to which H-NS-mediated silencing contributes to this cell-to-cell variation has remained unclear. Recent studies identified transcription-induced positive DNA supercoiling as a novel countersilencing mechanism, finding that positive supercoils generated by spurious transcription upstream of the SPI-1 master regulator *hilD* can disrupt nearby H-NS nucleoprotein complexes and contribute to the generation of SPI-1 bistability [16,17]. Determining how H-NS silencing contributes to virulence gene expression heterogeneity is therefore critical to understand how bistability is generated and maintained.

While the role of H-NS in virulence gene expression heterogeneity remains unclear, it is well established that H-NS facilitates pathogen evolution through its role as a xenogeneic silencer [1–3,36–38]. H-NS-mediated silencing is essential for maintaining virulence genes in the STm genome; without this tight silencing, the fitness costs of misexpressing virulence regulons lead to severe growth defects that are eventually compensated for by the loss of these regulons [6,36]. By immediately silencing newly acquired genes, H-NS acts as an evolutionary buffer, enabling the pathogen to retain these genes until it can integrate specific countersilencing mechanisms to precisely control their expression. The H-NS DBD is highly conserved, suggesting strong purifying selection against mutations that impair H-NS’s core silencing function [39]. While the evolutionary consequences of the complete loss of *hns* have been well characterized and primarily involve the deletion of the SPI-1 regulon, the consequences of subtle impairments in H-NS function on pathogen adaptation are less clear [36].

Here, we identify an STm *hns* hypomorph strain with a mutation in the DBD and use it to investigate how impairing this xenogeneic silencer alters phenotypic heterogeneity in virulence gene expression and evolutionary adaptation. By measuring virulence gene expression in individual bacteria, we find that impaired H-NS DNA-binding affinity increases the proportion of cells expressing virulence genes, enhancing epithelial cell infection. Using experimental evolution in intracellular-like conditions, we demonstrate that while both wild-type and mutant populations improve fitness by disrupting the SPI-2 virulence regulon, the mutant also acquires distinct adaptive mutations to resolve the underlying dysregulation in gene expression. These results suggest that even subtle impairments to H-NS’s silencing function have wide-ranging consequences, altering virulence activation thresholds in individual cells and the mutations acquired during adaptation.

## Results

### Identification of a *Salmonella* strain with a mutation in the H-NS DNA-binding domain

**Fig 1:**
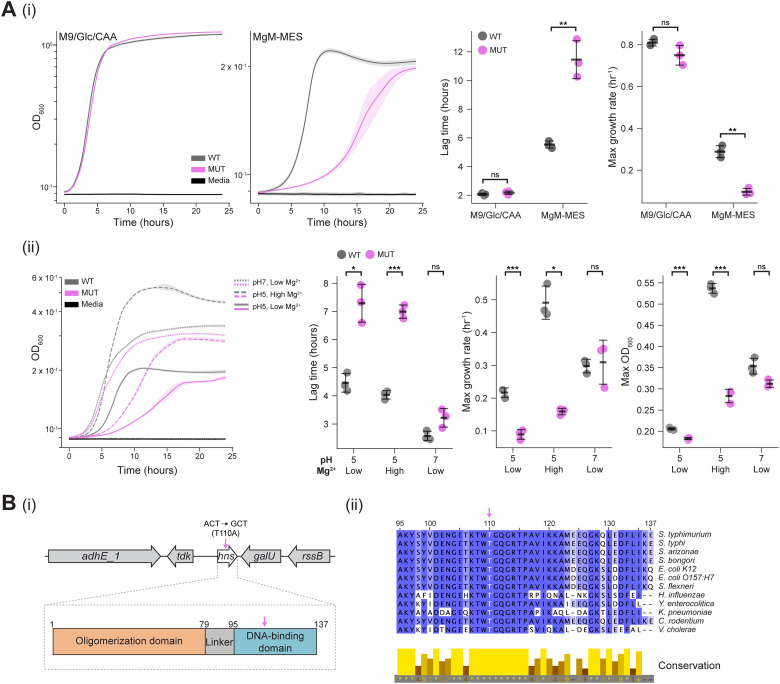
Identification of an H-NS DNA-binding domain mutation in a *Salmonella* strain with impaired growth in SPI-2-inducing conditions. **(A) (i)** The mutant strain exhibits growth defects in SPI-2-inducing conditions. Wild-type (WT, gray) and mutant (MUT, magenta) strains were grown in non-SPI-2-inducing media (M9/Glc/CAA) or SPI-2-inducing media (MgM-MES). OD_600_ was recorded using a plate reader and summary growth metrics (lag time, max growth rate) extracted. Growth curves show averages of three independent experiments, each with three technical replicates; shaded regions represent standard deviation. Media control is shown in black. Y-axes are independently scaled for each growth condition. For summary growth metrics, individual datapoints represent independent experiments. Asterisks denote significance as determined by two-sided unpaired t-test with Benjamini-Hochberg FDR correction as follows: ** p < 0.01; ns, not significant. **(ii)** Comparison of WT and MUT growth in media with varied pH or Mg^2+^ concentration. Growth curves show data from a single experiment, with three technical replicates. Summary metrics (lag time, max growth rate, max OD_600_) are displayed as in **(i)**. Asterisks denote significance as determined by Welch’s two-sided unpaired t-test with Benjamini-Hochberg FDR correction as follows: *p < 0.05, ***p < 0.001; ns, not significant. **(B)** The MUT strain contains a mutation in the conserved DNA-binding domain (DBD) of H-NS. **(i)** Schematic showing the genomic location of *hns* in STm and domain organization of the H-NS protein. Arrow indicates the location of the mutation identified by whole genome sequencing. **(ii)** Multiple sequence alignment showing conservation of the mutated residue (T110, white) across enteric bacteria. Dark blue and asterisks indicate complete conservation.

We obtained *Salmonella enterica* serovar Typhimurium (STm) 14028 from the ATCC repository and prepared glycerol stocks (see Methods). In SPI-2-inducing conditions (MgM-MES: pH 5, 8 µM Mg^2+^), we unexpectedly observed impaired growth relative to an STm wild-type (WT) reference strain in the lab, whereas growth in non-SPI-2-inducing conditions (M9/Glc/CAA) was unaffected (Fig 1A). The ATCC strain had a significantly increased lag time and a reduced maximum growth rate compared to the WT strain in MgM-MES (Fig 1A); we therefore subsequently refer to it as a mutant (MUT) strain. To assess whether acidic pH or limited magnesium was responsible for the growth defect in MgM-MES, we varied each signal independently and found that acidic pH was the major cause of the growth defect in the MUT strain (Fig 1A). To determine whether the impaired growth of the MUT strain in MgM-MES was generalizable to other virulence-inducing conditions, we cultured the strains in media used to induce the SPI-1 virulence regulon (LB High Salt: LB-HS) and found growth was comparable between the two strains (S1A Fig). Since MgM-MES imposes several stressors on the bacteria, including magnesium restriction, nutrient limitation, and acidic pH, we tested whether stress conditions were sufficient to elicit a growth defect in the MUT strain. The MUT strain showed impaired growth in a nutrient-limited condition (M9/Glc without casamino acids) compared to the WT strain, with significant changes in lag time and maximum growth rate (S1A Fig). Together, these data demonstrate that the MUT strain has context-specific growth defects, with impaired growth in SPI-2-inducing and nutrient-limiting media.

To identify the genetic basis for this context-specific growth defect, we performed whole genome sequencing on the WT and MUT strains. We identified a single missense mutation (a328g [T110A]) in the DNA-binding domain (DBD) of *hns* (Figs 1B, S1B). The H-NS DBD is highly conserved across enteric bacteria, with Thr110 completely conserved (Fig 1B) [39,40]. While several other genetic changes were identified in the MUT strain (S1B Fig, S1 Table), these were located in intergenic regions, a pseudogene, or in regions coding for hypothetical proteins, and were therefore considered unlikely to explain the growth defect. To confirm that the *hns* mutation was sufficient to cause the MgM-MES growth defect, we introduced the mutation into the WT strain (WT [T110A]) and reverted it in the MUT strain (MUT [A110T]). Growth depended on the *hns* genotype: the WT and MUT [A110T] strains grew similarly, as did the MUT and WT [T110A] strains (S1C Fig). In STm, *hns* null mutations are viable only when accompanied by mutations in *rpoS* or *phoP* [6,7,20]; therefore, this suggests that T110A is a hypomorph that impairs but does not abolish H-NS silencing of horizontally acquired genes.

### H-NS T110A has reduced DNA-binding affinity

**Fig 2:**
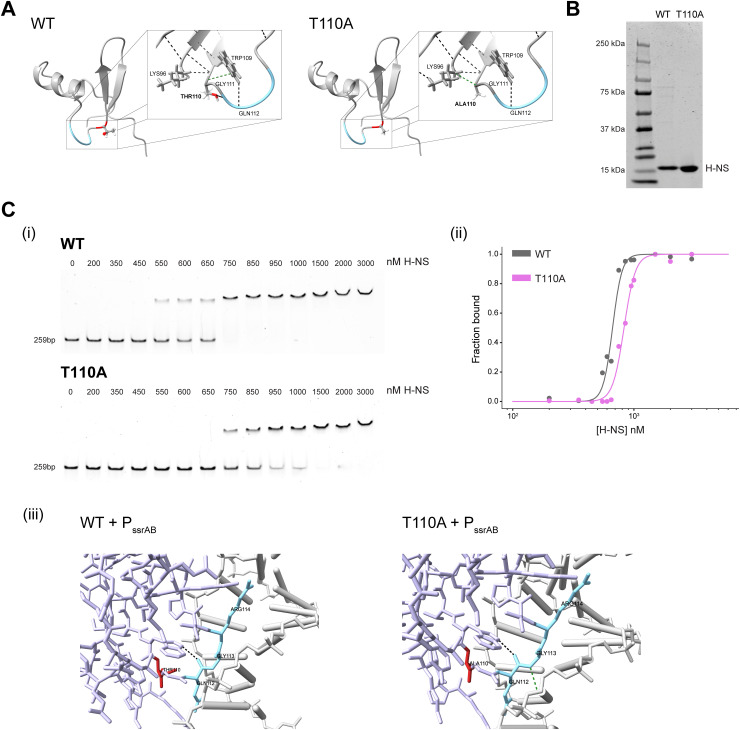
H-NS T110A has reduced DNA-binding affinity compared to H-NS WT. **(A)** Structure of STm H-NS WT and T110A DNA-binding domains modeled in ChimeraX using the NMR structure (PDB: 2L93). The T110A mutation is predicted to disrupt a polar contact (black dashed line) between the Thr110 side chain oxygen and the Gln112 backbone nitrogen. Gln112 is part of the QGR motif (shown in blue), an AT-hook that mediates direct DNA contacts. The mutation is also predicted to disrupt van der Waals interactions (green dashed line) between Thr110 and Trp109, while introducing a new interaction between Ala110 and Lys96. These structural changes may alter the positioning of the QGR motif and reduce its ability to bind the DNA minor groove. **(B)** Purified H-NS WT and T110A proteins analyzed by SDS-PAGE. Protein was visualized using Coomassie staining. **(C)** EMSAs demonstrate reduced DNA-binding affinity of H-NS T110A. **(i)** H-NS WT and T110A proteins were incubated with *ssrAB* promoter DNA at the indicated concentrations for 30 minutes and separated on a 5% native TBE gel. DNA was visualized using SYBR Safe staining. Representative gels are shown for WT (top) and T110A (bottom). **(ii)** Quantification of EMSA data from panel (i) showing the fraction of DNA-bound H-NS as a function of protein concentration. WT (gray) and T110A (magenta) data points are shown as circles. Lines represent fits to the Hill equation. **(iii)** Predicted interaction of H-NS WT and T110A (lavender) with the *ssrAB* promoter region (gray; as used in the EMSA) modeled using AlphaFold3 (random seed = 2484837). The region encompassing residue 110 (red) and the adjacent QGR motif (residues 112-114; blue) is shown. Predicted hydrogen bonds identified using ChimeraX are shown as dashed lines (black indicates a bond predicted in both the WT and T110A models, while green indicates a bond unique to the T110A model).

Based on the location of the T110A mutation in the highly conserved H-NS DBD and the T110A strain’s growth defects, we hypothesized that the mutation reduces H-NS DNA-binding affinity. To assess how the T110A mutation might alter protein structure, we used an NMR-resolved structure of the H-NS WT DBD from STm (PDB: 2L93) (Fig 2A) [8]. Two consequences of the T110A mutation that could negatively impact DNA binding were apparent. First, in the WT structure, Thr110 uses a side chain hydroxyl to form a polar interaction with Gln112, part of the QGR motif that mediates direct contacts between H-NS and the DNA minor groove [8,41]. The substitution of Thr110 with alanine eliminates the side chain hydroxyl and a methyl group, resulting in the loss of the stabilizing hydrogen bond between residues 110 and 112. Removing this polar interaction could destabilize the QGR motif and reduce H-NS DNA-binding affinity. Second, the T110A mutation alters van der Waals forces. In H-NS T110A, an interaction between Thr110 and Trp109 is lost, and a new interaction between Ala110 and Lys96 is gained. The Thr110 - Trp109 interaction likely anchors the polar bond between Trp109 and Gln112 to stabilize the QGR motif. This structural analysis confirms that Thr110 is a critical residue in the H-NS DBD and suggests that the T110A mutation disrupts key interactions that are important for proper positioning of the QGR motif.

Next, to test whether the T110A mutation reduces H-NS DNA-binding affinity, we performed electrophoretic mobility shift assays (EMSAs). We expressed and purified C-terminal 6xHis-tagged full-length H-NS WT and T110A in *E. coli* (Figs 2B, S2A-S2B, Methods). We selected a region in the *ssrAB* promoter as the DNA probe because *ssrAB* is a known H-NS target and this promoter region has been previously validated in H-NS EMSAs (S2C Fig) [14,42,43]. As predicted from the structural analysis, H-NS T110A retained DNA-binding capacity but had reduced affinity compared to H-NS WT (Figs 2C, S2D–S2E). Although there was some variation between replicates, the reduced affinity of H-NS T110A for DNA was reproducible. Free DNA probe was fully depleted between 750–950 nM for H-NS WT compared to 1500–2000 nM for H-NS T110A. We used AlphaFold3 to further explore the interaction of H-NS WT and T110A with the *ssrAB* promoter region used in the EMSA (see Methods) [44]. Half of the models predicted that the T110A mutation introduces an additional hydrogen bond within the QGR motif (Figs 2C, S2F). This additional bond may stabilize the local structure and reduce the conformational flexibility needed for binding DNA, potentially explaining the reduced DNA-binding affinity of T110A observed in the EMSAs. Together, these results demonstrate that the T110A mutation impairs H-NS function by reducing DNA-binding affinity, consistent with findings for *E. coli* H-NS DBD mutants [45,46].

### H-NS T110A alters heterogeneity in virulence gene expression

**Fig 3:**
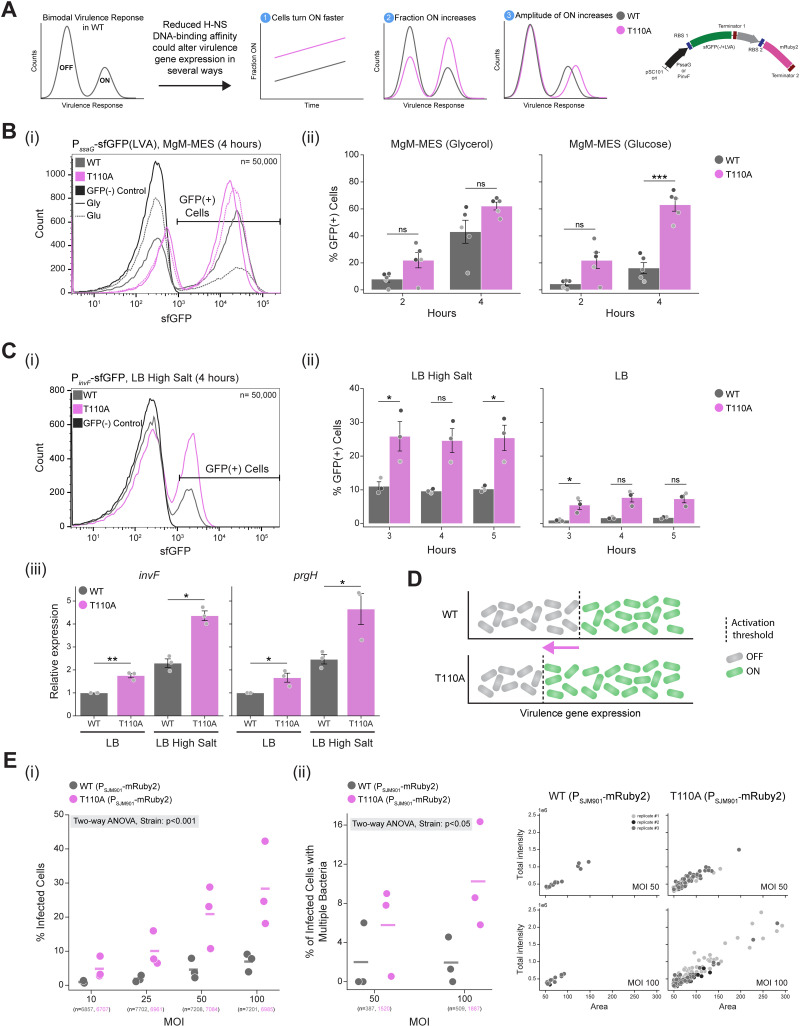
H-NS T110A increases the fraction of virulence-gene expressing cells with functional consequences for infection. **(A)** Schematics illustrating three potential outcomes of reduced H-NS DNA-binding affinity on virulence gene expression heterogeneity and a SPI-1/SPI-2 transcriptional reporter plasmid. **(B)** H-NS T110A cells activate the SPI-2 reporter more rapidly and with increased frequency, while maintaining bimodal expression. WT and T110A strains containing the SPI-2 reporter plasmid (P_*ssaG*_-sfGFP(LVA)) were grown in SPI-2-inducing media (MgM-MES) and analyzed by flow cytometry. **(i)** Representative histograms at 4 hours of WT (gray) and T110A (magenta) strains grown in MgM-MES with glycerol (solid line) or glucose (dashed line). GFP(-) control strain is shown in black. **(ii)** Quantification shows the percentage of GFP(+) cells at 2 and 4 hours. Bars represent the mean ± SD of 5 independent experiments. Individual datapoints are shown in shades of gray. n = 50,000 cells per sample. **(C)** H-NS T110A increases the frequency of SPI-1 responders while maintaining bimodal expression. WT and T110A strains containing the SPI-1 reporter plasmid (P_*invF*_-sfGFP) were grown in SPI-1-inducing media (LB High Salt) and non-inducing media (LB) and analyzed by flow cytometry. **(i)** Representative histograms at 4 hours of WT (gray) and T110A (magenta) strains grown in LB High Salt. GFP(-) control strain is shown in black. **(ii)** Quantification shows the percentage of GFP(+) cells at 3, 4, and 5 hours. Bars represent the mean ± SD of 3 independent experiments. Individual datapoints are shown in shades of gray. n = 50,000 cells per sample. For **(B)** (ii) and **(C)** (ii) asterisks denote significance as determined by linear mixed-effects model with post-hoc pairwise comparisons using unpaired t-tests with Holm-Sidak correction for multiple comparisons: * p < 0.05, *** p < 0.001; ns, not significant. (iii) qRT-PCR for *invF* and *prgH* in WT (gray) and T110A (magenta) strains after 4 hours of growth in LB High Salt or LB. For each gene, expression is shown relative to that of the WT strain in LB. Bars represent the mean ± SD of 3 independent experiments. Individual datapoints are shown as gray circles. Asterisks denote significance as determined by Welch’s t-test with Holm-Sidak correction for multiple comparisons: * p < 0.05, ** p < 0.01. **(D)** Activation threshold model for how H-NS alters the fraction of cells expressing virulence genes. In the T110A strain, the virulence activation threshold (dotted line) is lowered (pink arrow), increasing the responder fraction (green) while preserving bimodality. **(E)** H-NS T110A strain enhances epithelial cell infection. H-NS WT and T110A strains expressing constitutive mRuby2 (P_SJM901_-mRuby2) were used to infect HeLa cells at varying multiplicity of infection (MOI), and infections were imaged and quantified as described in the Methods. **(i)** Percentage of infected cells. Line represents the mean and circles the individual datapoints from 3 independent experiments. T110A results in an increased frequency of infected cells compared to WT (two-way ANOVA, effect of strain: F(1,16)=25.1, p < 0.001; n = total cells across 3 independent experiments). **(ii)** Percentage of infected cells that contain multiple bacteria. Line represents the mean and circles the individual datapoints from 3 independent experiments. T110A shows an increased frequency of multi-bacteria infection events (two-way ANOVA, effect of strain: F(1,8)=6.4, p < 0.05; n = total infected cells across 3 independent experiments). Scatter plot shows total intensity versus area of bacterial masks for infected cells containing multiple bacteria. Individual infected cells are represented as circles, and replicates are shown in shades of gray.

Virulence gene expression in *Salmonella* is heterogeneous, with only a subset of cells responding even under uniform virulence-inducing conditions [26–30,34,35]. Bimodality in virulence gene expression typically arises from stochastic gene expression and feedback in the gene regulatory network (GRN), generating subpopulations of responders and non-responders [47–50]. H-NS plays a critical role in silencing virulence genes in non-inducing conditions [6,7]; however, the extent to which H-NS contributes to heterogeneity in virulence gene expression remains unclear. To investigate this, we hypothesized that reduced H-NS DNA-binding affinity in the T110A strain could affect virulence heterogeneity in several distinct ways, which can be distinguished at the single-cell level (Fig 3A). For instance, in the T110A strain, 1) the response could be faster if H-NS is displaced more easily, 2) the fraction of responders could increase if H-NS displacement is a limiting step in expression, or 3) the amplitude of expression could increase if H-NS rebinding limits transcriptional output.

Given the impaired growth of the T110A strain in SPI-2-inducing media (MgM-MES, Fig 1A), we first examined whether the mutation altered SPI-2 gene expression patterns. To measure SPI-2 transcriptional activity in individual bacteria, we used a previously described transcriptional reporter consisting of the *ssaG* promoter fused upstream of a destabilized sfGFP (sfGFP-LVA) (Fig 3A) [30]. The reporter plasmid also constitutively expresses mRuby2 for cell identification by flow cytometry. In the WT strain grown in MgM-MES, SPI-2 reporter expression is bimodal, with bacteria progressively switching into the SPI-2 ON state over several hours [30]. As previously described, bimodality was independent of plasmid copy number, as the mRuby2 signal was unimodal and uncorrelated with sfGFP at the single-cell level (S3A Fig). We compared SPI-2 reporter expression between the WT and T110A strains in either SPI-2-inducing (MgM-MES (Glycerol)) or non-inducing (M9/Glc/CAA) media after 2 and 4 hours of growth. In SPI-2-inducing media, the T110A strain maintained bimodal SPI-2 reporter expression with distinct ON and OFF populations (Fig 3B). We next evaluated whether features of response heterogeneity (response timing, fraction of responders, and response amplitude) differed between the strains. To evaluate response timing and the fraction of responders, we compared the fraction of GFP(+) cells over time. At both 2 and 4 hours in SPI-2-inducing media, the T110A strain consistently had a greater fraction of responders than the WT strain (Fig 3B, S2 Table, 5/5 replicates, sign test, p = 0.0312). The increase in the fraction of GFP(+) cells at 2 hours, when cells first begin to express the SPI-2 reporter [30], indicates that SPI-2 reporter expression is faster in the T110A strain. To determine whether this increase in responder fraction over time in the T110A strain was a result of its slower growth rate in MgM-MES and protein accumulation, we substituted glucose for glycerol to increase the growth rate. At 4hrs, faster growth decreased the fraction of responders in the WT but not in the T110A strain, indicating that differences in growth rate cannot fully explain the increase in responders in the T110A strain (Figs 3B, S3B). The amplitude of the SPI-2 reporter response was generally similar between the two strains (S3C Fig) and as expected, under non-inducing conditions, the reporter remained off in both strains (S3D Fig), indicating that reduced H-NS DNA-binding affinity alone is insufficient to trigger SPI-2 expression in the absence of activated response regulators. Together, these data demonstrate that reduced H-NS DNA-binding affinity in the T110A strain increases the SPI-2 response rate and the fraction of responders.

Having established the effects of H-NS T110A on SPI-2 gene expression, we next examined SPI-1. SPI-1 expression imposes clear fitness costs, including reduced growth rate and increased antibiotic resistance [26,27]. In addition, the ratio of SPI-1(+) to SPI-1(-) cells is tightly regulated - mutations that decrease the fraction of SPI-1(-) cells lead to the emergence of genetically avirulent cells and limit infection [29]. Therefore, we hypothesized that the fraction of SPI-1(+) cells would be particularly sensitive to perturbations in H-NS-DNA binding affinity. To test this, we generated a SPI-1 transcriptional reporter by fusing the *invF* promoter upstream of sfGFP, with constitutive mRuby2 expression for cell identification by flow cytometry (Fig 3A) [51]. We examined reporter expression in SPI-1-inducing conditions (LB High Salt) and quantified the fraction of responders at 3, 4, and 5 hours by flow cytometry (Fig 3C). The T110A strain maintained bimodal SPI-1 reporter expression but had a higher fraction of responders than the WT, and this difference remained stable over time (Fig 3C, S2 Table). Bimodality was independent of plasmid copy number, and the amplitude of the response was similar between strains (S3E–S3F Fig). As expected, under non-inducing conditions (LB), the majority of cells remained OFF in the WT strain (Figs 3C, S3G). In contrast, the T110A strain showed a small but consistent increase in the fraction of SPI-1(+) cells relative to the WT. Neither strain had reporter expression prior to the start of the assay, indicating this difference arose during the experimental growth period (S3G Fig). Since WT and T110A strains grow similarly in LB (S1A Fig), growth differences cannot account for this increase in SPI-1(+) cells.

To confirm that our SPI-1 reporter reflected differences in endogenous SPI-1 gene expression, we performed qRT-PCR for two SPI-1 genes, *invF* and *prgH*, in WT and T110A strains without the reporter. Consistent with the reporter data, the T110A strain expressed both genes at higher levels than the WT in both LB High Salt and LB (Fig 3C). Finally, to demonstrate that the *hns* mutation alone was sufficient to increase the fraction of SPI-1(+) cells, we compared reporter induction across the WT, T110A, knock-in (WT [T110A]) and revertant (MUT [A110T]) strains after 4 hours in LB High Salt and LB (S3H Fig). The SPI-1 response tracked with the *hns* genotype. The fraction of responders was similar between WT and MUT [A110T] and between T110A and WT [T110A].

Together, these data suggest that the activation threshold for SPI-1 virulence gene expression is sensitive to changes in H-NS DNA-binding affinity (Fig 3D). In the T110A strain, lowering the SPI-1 activation threshold allows more cells to become SPI-1(+), while other known regulatory mechanisms maintain bimodality.

### Reduced H-NS function enhances *Salmonella* infection of epithelial cells

Expression of the SPI-1 regulon drives production of a T3SS required for invasion of epithelial cells [25,52,53]. However, increased SPI-1 reporter activity does not necessarily predict enhanced invasion. T3SS assembly requires numerous steps downstream of *invF* expression [54], and in other regulatory networks, distal genes can exhibit variable expression despite homogeneous expression of a proximal regulator [55]. In addition, invasion can also be impacted by the ratio of SPI-1(+) to SPI-1(-) cells. The presence of both subpopulations maximizes the invasion frequency of epithelial cells in culture, while a SPI-1(+) only subpopulation shows reduced invasion [56]. To test whether the increased fraction of SPI-1(+) cells in the T110A strain enhances epithelial cell infection, we infected HeLa cells with H-NS WT and T110A strains that constitutively express mRuby2 and quantified infection using microscopy. The frequency of infected HeLa cells was higher for the T110A strain than the WT across all multiplicities of infections (MOIs) tested (Fig 3E, S2 Table). Since this assay does not rely on a transcriptional reporter, it provides additional evidence that endogenous SPI-1 expression is increased in the T110A strain. We further characterized infection by quantifying the fraction of infected HeLa cells containing multiple bacteria, using area and total intensity of segmented bacterial objects to distinguish between single and multi-bacteria infection events. At higher MOIs (50, 100), where multi-bacteria infection events are more likely to occur, the frequency of such events was higher for the T110A strain than the WT, as was the number of bacteria per cell, as evidenced by greater area and total intensity of segmented bacteria (Fig 3E). Differences in bacterial replication cannot account for this effect, as bacteria were quantified shortly after infection, before intracellular replication had begun. Instead, the increase in multi-bacteria infection events in the T110A strain likely results from both the increased fraction of SPI-1(+) cells and the associated enhancement of membrane ruffling of epithelial cells, which has been shown to stimulate uptake of SPI-1(+) and nearby SPI-1(-) cells [57]. Together, these results demonstrate that reduced H-NS DNA-binding affinity directly affects epithelial cell infection.

### Impaired H-NS silencing alters pathogen adaptation under SPI-2-inducing conditions

H-NS buffers the fitness cost of horizontally acquired genes, silencing them to avoid the cost of inappropriate expression [1,2,36]. Disrupting this buffering capacity can be particularly deleterious, as even a single amino acid change can have widespread effects on gene expression. For instance, the T110A strain shows fitness defects in SPI-2-inducing and nutrient-limited media (Figs 1A, S1A), as well as altered virulence gene expression (Fig 3B-3C). We hypothesized that when the buffering capacity of H-NS is compromised, this would alter which mutations provide fitness benefits. In the WT background, where H-NS silencing has been optimized over evolutionary timescales, there may be limited capacity for fitness gains. In contrast, in the T110A background, H-NS function is impaired, and horizontally acquired genes are misregulated. Therefore, adaptation is expected to favor compensatory mutations, such as those that reduce the expression of misregulated genes or restore silencing activity, to overcome the associated fitness defects. While the consequences of a complete loss of H-NS buffering capacity on pathogen adaptation have been well described and involve loss of SPI-1, comparing the evolutionary trajectories of the H-NS WT and T110A strains allowed us to assess how a more subtle disruption of this buffering capacity affects pathogen adaptation [36].

**Fig 4:**
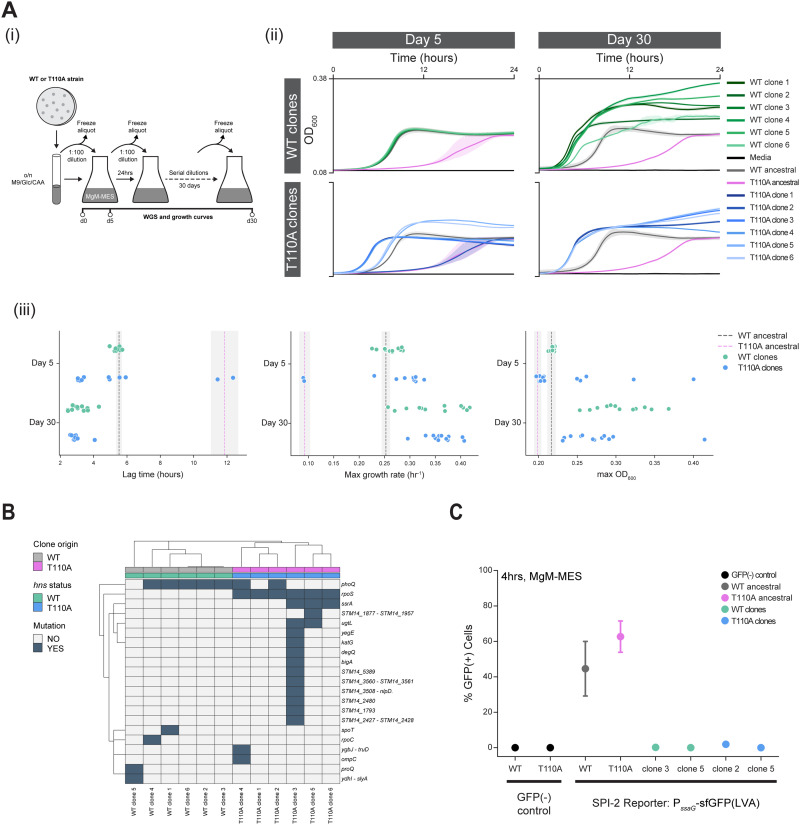
Impaired H-NS silencing alters adaptive mutations that improve fitness under SPI-2-inducing conditions. **(A)** Experimental evolution reveals fitness gains in both WT and T110A strains. **(i)** Schematic showing the experimental evolution approach. Six individual colonies from each of the WT and T110A strains were inoculated into M9/Glc/CAA overnight, then subcultured daily in 25 mL SPI-2-inducing media (MgM-MES) for 30 days. Glycerol stocks were made daily. Evolved clones at day 5 (d5) and day 30 (d30) were assessed for growth and genotype using plate reader assays and whole genome sequencing. **(ii)** Growth dynamics of evolved clones. Most T110A clones (shades of blue) show increased fitness by d5 that stabilizes by d30, while WT clones (shades of green) remain similar to the ancestral WT strain at d5 but show increased fitness by d30. Strains were grown in MgM-MES and OD_600_ was recorded using a plate reader. Growth curves show means of three technical replicates, and shaded regions represent standard deviation. Ancestral strains (WT - gray, T110A - magenta) and media control (black) are shown in each plot for comparison. Representative experiments for d5 and d30 are shown. **(iii)** Growth metrics extracted from data in (ii) and S4A Fig. Circles represent WT (green) and T110A (blue) evolved clones; dashed lines represent means of the ancestral WT and T110A strains; shading indicates variation between two replicates. **(B)** WT and T110A evolved clones acquire distinct mutations by d30. Genomic DNA from d30 evolved clones was sequenced and mutations are displayed as a clustered heatmap. WT and T110A clones cluster separately. Colorbars indicate ancestral strain (WT - gray, T110A - magenta) and *hns* genotype at d30 (WT - green, T110A - blue). Dark blue in the heatmap indicates the presence of a mutation in the indicated gene, while light gray indicates no mutation. If a large deletion includes a gene shown elsewhere on the heatmap, that gene is shown in dark blue. **(C)** Evolved clones show negligible SPI-2 induction. SPI-2 induction was assessed in two d30 clones from each background (WT clones 3 and 5; T110A clones 2 and 5) using the SPI-2 GFP reporter plasmid (P_*ssaG*_-sfGFP(LVA)). Samples were analyzed by flow cytometry after 4 hours in MgM-MES and the percentage of GFP(+) cells compared to that in the ancestral WT and T110A strains. Ancestral strains with GFP(-) control plasmids are shown as a reference for background fluorescence values.

We subjected six independent cultures of each strain to experimental evolution in SPI-2-inducing media (MgM-MES) for 30 days (Fig 4A) and compared growth metrics, genomic changes, and virulence gene expression. WT and T110A evolved clones displayed distinct growth dynamics (Figs 4A, S4A). At day 5, the WT clones showed no improvement compared to the ancestral WT strain. In contrast, five of six T110A strains had improved growth and surpassed even the ancestral WT strain, with variable changes in lag time, maximum growth rate, and maximum OD_600_. By day 30, all WT and T110A clones had improved growth relative to the ancestral WT strain across all growth metrics, though the magnitude of improvement varied among clones. The differences in the timing of fitness improvements (faster adaptation for T110A) suggest that distinct mutational trajectories occur in each *hns* genotype and that the T110A strain is genetically unstable, similar to Δ*hns* strains [36].

To identify strain-specific adaptive mutations, we performed whole-genome sequencing on all day 30 evolved clones (Figs 4B, S4B). WT and T110A clones acquired distinct sets of mutations, as shown by hierarchical clustering. Revertants restoring H-NS T110A to WT were never observed. Instead, all T110A clones acquired compensatory mutations in *rpoS,* a stress response sigma factor, and 5/6 acquired mutations in *ssrA* (a sensor kinase that activates the SPI-2 master regulator SsrB) or *phoQ* (a sensor kinase that activates SPI-2 in response to acidic pH and limited magnesium) [20,58–62]. The prevalence of *rpoS* mutations is consistent both with H-NS promoting RpoS degradation and STm Δ*hns* strains acquiring compensatory mutations in *rpoS* [6,7,63]. One T110A clone (clone 3) acquired a mutation in *mutS*, a mismatch repair gene, resulting in numerous additional mutations [64]. In contrast to the T110A clones, all WT clones acquired mutations related to the PhoPQ two-component system. Five clones carried mutations in the sensor kinase *phoQ*, while one carried a mutation in *proQ*, an RNA-binding protein that positively regulates the PhoP regulon [65,66].

Sequencing of three day 5 T110A clones revealed few mutations, consistent with these populations likely being a mixture of cells carrying different mutations that had yet to become fixed in the population (S4B Fig). To evaluate the extent of genetic variation within the day 30 clones, we sequenced two additional single colonies from a subset of WT and T110A clones (S4C Fig). For the WT clones, additional colonies were either genetically identical to the original (clone 1), differed only in the specific *phoQ* residue mutated (clones 2, 6), or carried mutations in different genes (clone 5). In contrast, the T110A clones showed greater within-clone variability in the specific residues or genes mutated. Yet the majority of T110A colonies (11/12) contained a mutation in *rpoS* or *nlpD*, an upstream gene with regulatory elements for *rpoS*, reinforcing *rpoS* as the primary target for adaptation in the T110A genetic background [67]. These data show that despite greater within-clone variation in T110A, mutations converge on *rpoS* and the SPI-2 GRN (*ssrA*, *ssrB*, *phoQ*). In terms of growth, the single colonies for each clone showed similar lag times and maximum growth rates as the bulk population, though maximum OD_600_ was variable (S4C Fig). This may reflect underlying genetic variation, as genetically identical colonies generally showed similar maximum OD_600_ values (WT clones 1 and 6 and T110A clone 4).

To assess whether these mutations in the SPI-2 GRN altered virulence gene expression, we measured SPI-2 reporter expression in two representative day 30 clones from each background (WT clones 3 and 5, T110A clones 2 and 5). After 4 hours in MgM-MES, all four clones showed negligible GFP(+) cells by flow cytometry (Figs 4C, S4D). In three of the clones, the small fraction of GFP(+) cells had intensity measurements similar to those of rare cells in the GFP(-) control strain labeled as GFP(+), confirming that these cells are unlikely to be SPI-2 responders (S4D Fig). Only T110A clone 2 had GFP(+) cells of similar intensity to the ancestral strains, indicating that this clone retained SPI-2 activity, albeit in a much decreased fraction of cells.

Overall, the genomic changes observed in the evolved clones demonstrate that while both strains adapt by silencing SPI-2 gene expression, the starting *hns* genotype dictates the specific adaptive mutations. While the WT clones acquire mutations in PhoPQ, the T110A clones acquire two distinct sets of mutations: *rpoS* mutations to alleviate the dysregulation of horizontally acquired genes and mutations in the SPI-2 GRN. Together, these results reveal how impaired H-NS silencing alters pathogen adaptation to intracellular-like environments.

## Discussion

Here, we identified an STm *hns* hypomorph strain and used it to examine the contribution of H-NS-mediated silencing to virulence gene expression heterogeneity and pathogen adaptation. Biochemical characterization confirmed that the T110A mutation reduced H-NS DNA-binding affinity *in vitro*. Using SPI-1 and SPI-2 transcriptional reporters, we found that reduced H-NS DNA-binding affinity increased the proportion of responding cells while preserving bimodal reporter expression. The increased fraction of SPI-1(+) cells in the T110A strain had functional consequences, resulting in greater infection of epithelial cells *in vitro*. Finally, using experimental evolution, we demonstrate that distinct adaptive mutations are associated with fitness gains in the WT and T110A strains, likely because the T110A strain must resolve its underlying dysregulation in gene expression. Together, our findings establish that the xenogeneic silencer H-NS contributes to virulence gene expression heterogeneity by setting virulence activation thresholds, and that even minor impairments of H-NS function alter the mutations acquired during adaptation to intracellular-like environments.

The critical role of H-NS in silencing virulence gene expression in non-inducing environments has been well-established using Δ*hns* strains, while elegant biochemical studies have characterized the role of DNA-binding proteins, including response regulators, in displacing H-NS from virulence gene promoters [6,7,13,14,36]. However, the contribution of H-NS-mediated silencing to heterogeneity in virulence gene expression has remained unclear for several reasons. First, evaluating the role of a global silencer like H-NS in virulence heterogeneity is challenging because Δ*hns* strains have widespread dysregulation of horizontally acquired genes and thus exhibit severe growth defects even in rich media, rapidly selecting for compensatory mutations that confound the interpretation of H-NS’s contribution to any phenotype [6,7,36]. Second, in Δ*hns* strains, several STm virulence regulons are broadly expressed in non-inducing environments, precluding analysis of how countersilencing mechanisms impact virulence heterogeneity in inducing conditions [6,7]. Because the H-NS T110A strain grows normally under non-inducing conditions, thereby limiting selective pressure for compensatory mutations, while maintaining baseline silencing of virulence genes, it is ideally suited to examine H-NS’s role in generating virulence heterogeneity.

Horizontally acquired DNA is typically AT-rich, supporting high baseline levels of transcription in the absence of H-NS [68]. Because H-NS silencing relies on oligomerization and higher-order complex formation, it functions as an inherently digital switch. Therefore, the reduced H-NS DNA-binding affinity conferred by the T110A mutation would not be expected to cause a gradual, population-wide change in virulence gene expression. Instead, the mutant H-NS lowers the activation threshold required for a countersilencing mechanism to disrupt the nucleoprotein filament, changing the fraction of responders rather than individual expression levels. As such H-NS silencing and countersilencing may provide a mechanism for bet-hedging, enabling the pathogen to harness phenotypic heterogeneity to survive in host niches. Our finding that subtle changes in H-NS DNA binding affinity increase the proportion of virulence responders while maintaining bimodality aligns with recent work demonstrating that DNA supercoiling driven by spurious transcription can destabilize H-NS nucleoprotein filaments and contribute to SPI-1 bistability [16,17]. Together, these data indicate that H-NS plays a critical role in establishing single-cell virulence activation thresholds and that perturbations to H-NS silencing, whether through changes in DNA supercoiling or reduced H-NS DNA-binding affinity, can lower these thresholds and increase the fraction of responding cells. As virulence genes are a subset of horizontally acquired genes and H-NS silencing operates by the same mechanism regardless of gene function, we propose that H-NS may affect phenotypic heterogeneity more broadly, and that future studies should examine this across other horizontally acquired genes.

Two models could explain the increase in the proportion of virulence responders in the T110A strain: 1) response regulator levels vary continuously across the population, and in certain bacteria these levels are insufficient to displace H-NS WT but capable of displacing the lower affinity H-NS T110A; or 2) the density of H-NS silencing of virulence genes varies between individual bacteria, meaning that weakening H-NS binding affinity shifts only a subset of cells past the activation threshold. These models make distinct, testable predictions, although testing them will require technically challenging single-cell measurements, such as correlating levels of response regulators with virulence gene expression or quantifying cell-to-cell variation in H-NS deposition at specific virulence gene promoters.

STm has evolved to navigate fluctuating host environments by tuning virulence gene expression through the dynamic balance between H-NS silencing and countersilencing across different host niches [13,14,69–71]. Tracking these virulence responses in single cells over time has revealed critical features of the SPI-2 response, including switch-like activation and heterogeneity in response timing [30]. An important future extension of our findings will be to examine virulence gene expression over time in individual bacteria with either intact or impaired H-NS silencing, both *in vitro* and in host cells. The T110A mutation likely has phenotypic consequences that extend beyond the fraction of virulence responders, potentially altering the trajectory of the virulence response, its stability across generations, or its reversibility as cells transition to new environments. Such studies will be critical to gain further mechanistic insight into how H-NS-mediated silencing of virulence genes has been optimized over evolutionary time.

Our experimental evolution data highlight H-NS’s critical role in buffering the cost of horizontally acquired genes. While both WT and T110A strains converged on disrupting the SPI-2 regulatory network to improve fitness in intracellular-like conditions, mutations arose in different nodes in the network depending on the starting *hns* genotype, and all T110A clones acquired mutations in *rpoS*. This divergence in adaptive mutations indicates that the mutant likely has to compensate for dysregulated baseline gene expression. While a prior experimental evolution study using an *hns* null strain established that H-NS buffers the immediate fitness costs of horizontally acquired genes, specifically the SPI-1 regulon, the extent to which more subtle impairments in H-NS function affect pathogen adaptation was unclear [36]. Our results demonstrate that even minor, non-lethal perturbations to H-NS compromise its buffering capacity, altering the specific adaptive mutations that arise under virulence-inducing conditions. Finally, in both *hns* genotypes, adaptation came at a cost, the loss of SPI-2-inducing capacity. This indicates a tradeoff between fitness and maintenance of intact virulence gene regulation. Although technically challenging to implement, future experimental evolution approaches should incorporate periodic selection for functional SPI-2 to reveal whether bacterial populations can simultaneously optimize growth and maintain inducible virulence gene regulation, or whether this is a fundamental constraint that limits pathogen adaptation.

In summary, our data demonstrate that H-NS is a critical buffer of gene expression in STm, and even minor impairments in H-NS function are sufficient to disrupt single-cell virulence activation thresholds and alter the adaptive mutations that improve fitness.

## Methods

Essential reagents and catalog numbers are listed in S3 Table.

Raw gel images have been deposited on Zenodo at https://doi.org/10.5281/zenodo.20750401.

### *Salmonella* strains

The *Salmonella enterica* serovar Typhimurium 14028 strain used as wild type (WT) was a gift from the laboratory of Michael McClelland. The *hns* mutant strain was isolated from a freeze-dried stock of *Salmonella enterica* serovar Typhimurium 14028 obtained from ATCC (ATCC 14028). The strain was rehydrated and streaked onto nutrient agar according to ATCC protocols. Colonies from the initial streak varied in morphology, and several appeared mucoid. Individual colonies were restreaked onto LB agar plates and glycerol stocks prepared. One glycerol stock was used for all experiments and is referred to as the *hns* mutant or T110A strain throughout.

### *Salmonella* cell culture

Bacterial strains were streaked from glycerol stocks onto LB agar containing appropriate selective antibiotics and incubated overnight at 37˚C. Plates were used for experiments within one week and culture growth kept to a minimum to avoid issues of genomic instability associated with *hns* mutant strains [36]. Cultures were grown at 37˚C with shaking (250 rpm). Growth media compositions: LB-Lennox (LB): NaCl 5 g/L; LB-Miller (LB High Salt): NaCl 10 g/L (SPI-1-inducing media); M9/Glc/CAA: 1x M9 salts, 0.1 mM CaCl_2_, 2 mM MgSO_4_, 0.4% glucose, 0.2% casamino acids; M9/Glc: 1x M9 salts, 0.1 mM CaCl_2_, 2 mM MgSO_4_, 0.4% glucose; MgM-MES (SPI-2-inducing media): 5 mM KCl, 7.5 mM (NH_4_)_2_SO_4_, 0.5 mM K_2_SO_4_, 1 mM KH_2_PO_4_, 170 mM 2-(N-morpholino)ethanesulphonic acid hydrate (pH adjusted to 5.0 with NaOH), 0.1% casamino acids, 38 mM glycerol, 8 µM MgCl_2_ [72,73]. Where indicated for SPI-2-inducing media, glycerol was replaced with 0.4% glucose, magnesium concentration was adjusted from 8 µM to 2 mM, and MOPS buffer was used instead of MES for pH7 media.

### Plasmids, cloning, and *Salmonella* strain generation

All plasmids and strains generated in this study are listed in S4 Table. Plasmids were assembled using either Golden Gate Assembly with the EcoFlex library or Gibson assembly [74]. *E. coli* TG1 cells were used for all cloning steps. Plasmids were verified by Sanger sequencing or whole plasmid sequencing.

*SPI-1 reporter plasmid (P*_*invF*_*-sfGFP):* The *invF* promoter region (340 bp upstream of the start codon plus 179 bp of the coding region) was previously described [51] and was amplified from STm genomic DNA using PrimeSTAR Max DNA polymerase and cloned into a Golden Gate Level 0 promoter plasmid using Gibson assembly. A Level 1 single transcriptional unit (TU) plasmid containing the promoter, ribosome binding site, open reading frame, and terminator was assembled by Golden Gate Cloning. This plasmid was combined with a second TU expressing a constitutive mRuby2 to generate the final Level 2 plasmid.

*SPI-2 reporter plasmid (PssaG-sfGFP-LVA) and constitutive mRuby2 plasmid (P*_*SJM901*_*-mRuby2):* These plasmids were generated using the same strategy and have been previously described [30].

*Strain construction:* Electrocompetent cells were prepared using the mannitol-glycerol preparation method [75]. Reporter plasmids were used to transform bacteria, and transformants were selected on LB agar plates containing the appropriate antibiotic (see S4.2 Table). WT strains containing the SPI-2 reporter plasmid or the constitutive mRuby2 plasmid were previously generated [30]. Glycerol stocks were prepared by mixing 700 µl overnight culture (grown in LB with appropriate antibiotics) with 300 µl of 50% w/v glycerol, vortexing, and storing at -80˚C.

Lambda red recombineering was used to introduce the T110A mutation into the WT strain and to revert it in the T110A strain [76]. WT and T110A cells were transformed with pKD46, a plasmid that contains arabinose-inducible lambda red proteins and a temperature-sensitive origin of replication. Gibson assembly was used to create plasmids containing the *hns* 3’ coding region (WT or T110A) with a FRT-cat-FRT cassette inserted between *hns* and *galU* (S4.1 Table), and these were used to PCR amplify a repair template with 40 bp homology to the genome. Purified PCR products were transformed into arabinose-induced cells (WT and T110A) containing pKD46, and cells were grown for 4 hours at 37˚C before plating on LB/Chloramphenicol. Genome sequencing was used to verify the correct insertion of the mutation and selection cassette. To remove the FRT selection cassette, colonies were transformed with pCP20, a temperature-sensitive plasmid expressing Flpe recombinase. Single colonies were then grown at 30˚C for 2 hours, shifted to 42˚C for 5 hours to induce Flpe expression, then returned to 30˚C overnight [77]. Removal of the cassette was confirmed by the loss of chloramphenicol resistance.

### Mammalian cell culture

HeLa cells from ATCC were maintained in DMEM supplemented with 10% FBS, 1x Penicillin/Streptomycin, and 2 mM GlutaMAX at 37˚C, 5% CO_2_. Cell lines were not authenticated.

### Generation of stable cell lines

Lentivirus was produced in Lenti-X 293T cells using packaging plasmids psPAX2 (Addgene #12260) and pMD2.g (Addgene #12259). HeLa cells were transduced with lentivirus expressing H2B-miRFP670 and selected with hygromycin.

### Plate reader growth assays and analysis

Single colonies were inoculated into the appropriate pre-culture medium (M9/Glc/CAA for M9/Glc/CAA, M9/Glc, and MgM-MES plate reader samples and LB-Lennox for LB-Lennox and LB-Miller plate reader samples) and grown shaking at 37˚C for 14–16 hours. When a bulk population was used for comparison (S4C Fig), an overnight culture was inoculated using a toothpick scraped across the relevant plate. All overnight cultures were back diluted to OD_600_ of 0.06 in 3 mL of the same medium and grown shaking for 3 hours. Cells were pelleted, washed twice in the plate reader media, and diluted to OD_600_ of 0.002. Three technical replicates (150 µl each) were added to a glass-bottom 96-well black plate. Plates were sealed with breathable film, and a needle was used to pierce each well to ensure aeration. Following a 30-minute equilibration in the plate reader (Tecan Spark) at 37˚C, OD_600_ was measured at 5-minute intervals for 24 hours. Growth curve metrics (lag time and maximum growth rate) were extracted using the Omniplate software package [78]. Omniplate fits a Gaussian process to the OD_600_ timecourse data and estimates instantaneous growth rate as a function of time; the maximum growth rate is defined as the largest instantaneous value. Omniplate estimates lag time as the time at which the growth rate exceeded 1% of its maximum value. The maximum OD_600_ was found by smoothing the data and identifying the maximum OD_600_ over the timecourse. Plots were generated using Seaborn and Matplotlib in Python [79,80]. Statistical analyses were performed in Python (scipy and statsmodels) using unpaired t-tests. Asterisks denoting significance level are indicated in the Figure legends.

### Whole Genome Sequencing (WGS) and analysis

Strains were streaked onto LB agar plates and incubated overnight at 37˚C. Single colonies were inoculated into 3 mL MgM-MES and grown shaking overnight at 37˚C. Genomic DNA was isolated from 0.5-1 mL of culture using the Wizard(R) Genomic DNA purification kit (Promega) and eluted in Tris pH 8.5. DNA samples were processed for Illumina whole-genome sequencing (200 Mbp sequencing) by SeqCenter (https://www.seqcenter.com). The additional single colonies (S4C Fig) were sequenced by Plasmidsaurus.

Sequencing reads were analyzed using *breseq* [81]. FASTQ files were aligned to the STm 14028s reference genome, including the chromosome (GenBank: CP001363.1) and plasmid (GenBank: CP001362.1) sequence files. All samples were compared to the STm WT strain used in the lab. The mutation heatmap (Fig 4B) was generated in R using the pheatmap package with binary distance clustering (clustering_distance_cols = ‘binary’) and complete linkage (clustering_method = ‘complete’). A complete list of genetic changes for all WGS samples is reported in S1 Table.

### Protein sequence alignment

Protein sequences of H-NS homologs from Gram-negative enteric bacteria were retrieved from UniProt. VicH, an H-NS-like protein from *Vibrio cholerae*, was also included [82]. Sequences were aligned using Clustal Omega with output order set to match input order [83]. Alignments were visualized using Jalview with color set to percent identity and conservation scores displayed [84].

### Protein structure analysis

Structural analysis was performed in UCSF ChimeraX (version 1.10.1) using the NMR-resolved structure of the STm H-NS DNA-binding domain (PDB ID: 2L93) [8,85]. The T110A mutant model was generated by substituting alanine for threonine at position 110 in the wild-type structure. Wild-type and T110A models were compared to evaluate local structural effects of the substitution around the QGR motif. Hydrogen bonds were displayed, and van der Waals interactions were identified by displaying contacts within 3.5 Å.

AlphaFold 3 was used to explore the interaction of H-NS WT and T110A with the *ssrAB* promoter region (dsDNA). Ten different seeds were used to reduce bias. Hydrogen bonds in the AlphaFold-predicted structures were identified using UCSF ChimeraX (version 1.10.1) with default hydrogen-bond detection criteria. These interactions were visualized as pseudobonds and used for comparative analysis between the H-NS WT and T110A models.

### Protein expression and purification

Expression plasmids for H-NS WT and T110A were generated in the pET-28a backbone with a C-terminal PreScission protease (PPX) cleavage site and 6xHis tag (S2A Fig). Expression plasmids were used to transform competent *E. coli* BL21 (DE3) cells, and transformants were selected on LB agar with kanamycin (50 µg/mL). Cultures were grown in LB-Lennox with kanamycin to OD_600_ of 0.5, induced with 0.5 M isopropyl-beta-D-thiogalactopyranoside (IPTG), and grown for 2 hours at 37°C with shaking (250 rpm). Cells were pelleted (14,000 rpm, 30 minutes, 4°C), resuspended in lysis buffer (20 mM Tris-HCl pH 8, 150 mM NaCl, 5% v/v glycerol, 2 mM 1,4-dithiothreitol (DTT), 1 mM phenylmethylsulfonyl fluoride (PMSF), 1x EDTA-free protease inhibitor) and sonicated on ice (Branson, output control of 2.5%, 20% duty cycle, 5-second pulses with 45-second rest intervals on ice). Lysates were clarified by centrifugation (14,000 rpm, 30 minutes, 4°C). All subsequent precipitation steps were performed at 4°C. DNA was precipitated out of clarified lysates by adding polyethyleneimine (PEI; avg. MW 60 K) slowly and drop-wise: 0.4% PEI in 150 mM NaCl for H-NS WT, and 0.35% PEI in 1 M NaCl for H-NS T110A. Samples were incubated for 15 minutes with gentle stirring and then centrifuged (11,000*xg*, 15 minutes, 4°C). The supernatant was gently decanted into a beaker, and H-NS was salted out by slow addition of powdered ammonium sulfate to 37% saturation. The solution was stirred gently overnight, and precipitated H-NS was collected by centrifugation (15,000 rpm, 40 minutes, 4°C). The H-NS precipitate was carefully resuspended in 10 mL of binding buffer (20 mM Tris-HCl pH 8, 300 mM NaCl, 20 mM imidazole, 5 mM β-mercaptoethanol). Samples were applied to pre-equilibrated Ni-NTA agarose resin (Invitrogen) and incubated on ice with gentle rocking for 1 hour. Resin was pelleted (800*xg,* 1 minute, 4°C), and the flow-through was discarded. To reduce non-specific binding and disrupt remaining protein-DNA interactions, the resin was washed with wash buffer (20 mM Tris-HCl pH 8, 600 mM NaCl, 50 mM imidazole, 5 mM β-mercaptoethanol) for 1 hour on ice with gentle rocking, then pelleted (800*xg*, 1 minute, 4°C). Wash buffer was decanted, and cleavage buffer was added (20 mM Tris-HCl pH 8, 300 mM NaCl, 5 mM β-mercaptoethanol) along with PreScission Protease (1 µl per nmol H-NS). Samples were incubated on ice with gentle rocking for 4 hours. Resin was pelleted (800*xg*, 1 minute, 4°C) and the H-NS-containing flow-through was collected. Samples were concentrated using a 3kDa MWCO cutoff Ultra Centrifugal Filter (Amicon) and subjected to size-exclusion chromatography (SEC) on a Superdex 75 Increase 10/300 GL column (Cytiva) equilibrated in SEC buffer (20 mM Tris-HCl pH 8.0, 300 mM NaCl, 5% glycerol, 5 mM β-mercaptoethanol) and run at a flow rate of 0.8 mL/min on an ÄKTA Avant 25 system (GE Healthcare) at Northwestern University’s Biofoundry Core. Peak fractions were analyzed by Coomassie-stained SDS–PAGE and UV–Vis spectrophotometry. Protein purity was quantified from the Coomassie-stained gel using ImageJ [86] and determined to be ~ 90% for H-NS WT and 99% for H-NS T110A. Absorbances at 260 nm and 280 nm were used to verify protein concentration and absence of nucleic acids. A_260_/A_280_ ratios for all samples were approximately 0.6, consistent with minimal nucleic acid contamination. Samples were flash-frozen in liquid nitrogen and stored at -80°C.

### Electrophoretic Mobility Shift Assays (EMSAs) and analysis

A region of the *ssrAB* promoter region was amplified from STm genomic DNA using PrimeSTAR DNA Polymerase (Takara) with primers previously described (S2C Fig) [42]. PCR products were purified (Zymo Research) and verified by gel electrophoresis and Sanger sequencing (Azenta). H-NS WT and T110A proteins were exchanged into EMSA binding buffer (20 mM Tris-HCl pH 7.4, 4 mM MgCl_2_, 100 mM NaCl, 1 mM DTT) using 3kDa MWCO cutoff Ultra Centrifugal Filters (Amicon). Binding reactions were assembled in 0.2 mL reaction tubes. Water, then EMSA Binding Buffer was added to the desired volumes and mixed. Protein was added (0, 200, 350, 450, 550, 600, 650, 750, 850, 950 nM, 1, 1.5, 2, 3 µM) and gently mixed. In 30-second increments, 25 ng DNA probe per reaction tube was added. Samples were incubated at room temperature for 30 minutes. Loading dye (40% glycerol, 0.25% bromophenol blue) was added, and samples were loaded onto a 5% native Mini-PROTEAN TBE gel (Bio-Rad) in 30-second increments to maintain consistent timing across reactions. Gels were run at 100V in 0.5x TBE buffer for 30 minutes, stained with Sybr Safe (Invitrogen), and washed twice with 150 mL Milli-Q H_2_O. Gels were imaged on an Azure 600 (Azure Biosystems Inc.) with a 10-second exposure time.

Gels were quantified using ImageJ. Background subtraction was performed by measuring the median intensity of four background regions and calculating the mean. Bound and unbound DNA regions were defined for each lane on a gel using the ROI manager tool. The fraction bound was calculated for each lane as: fraction bound = (bound) / (bound + unbound), and a min-max normalization was performed. All calculations and non-linear regression fitting to the Hill equation were performed using SciPy and Python.

### SPI-1 and SPI-2 induction assays

*SPI-1 induction:* Single colonies were inoculated into 3 mL LB-Lennox with appropriate antibiotics and grown shaking at 37°C for 14–16 hours. Overnight cultures (BD1) were back diluted to OD_600_ of 0.06 in fresh LB-Lennox and grown shaking for 2.5 hours (BD2). Cultures were then back diluted to an OD_600_ of 0.06 into 5 mL LB-Miller (SPI-1-inducing) or LB-Lennox (control). At the indicated fixation timepoints, 600 µl of culture was mixed with 200 µl of 16% paraformaldehyde by inversion and incubated at room temperature for 30 minutes. Fixed samples were washed three times with 1 mL PBS before storing in 1 mL PBS at 4˚C. Samples were analyzed by flow cytometry within one week of fixation.

*SPI-2 induction:* Single colonies were inoculated into 3 mL M9/Glc/CAA with appropriate antibiotics and grown shaking at 37°C for 14–16 hours. Overnight cultures were back diluted 1:100 into 3 mL fresh M9/Glc/CAA and grown shaking for 4 hours. Cultures were then back diluted to an OD_600_ of 0.02 in 5 mL of MgM-MES containing either glycerol or glucose. Samples were fixed and stored as described for SPI-1 induction.

### Flow cytometry and analysis

Fixed samples were diluted in PBS to achieve approximately 10,000 events per second or below. Samples were analyzed on a BD LSRFortessa SORP Cell Analyzer with HTS (6-laser 18-parameter). At least 50,000 mRuby2 positive events were captured per sample.

Data were analyzed using FlowJo v10.8.2. Forward scatter (FSC) and side scatter (SSC) gates were drawn to capture the majority of events. All reporter strains constitutively express mRuby2; therefore, mRuby2 positive gates were set using negative control samples (cells without the mRuby2 plasmid) such that less than 0.05% of the negative control events were classified as mRuby2 positive. mRuby2 positive events were downsampled to 50,000 using the FlowJo Downsample plugin (V3.3.1) for all subsequent analyses. GFP-positive gates were set using a GFP-negative control sample (strain with a promoterless GFP control plasmid). Statistics, including mean fluorescence intensity (MFI) and percentage of GFP(-) and GFP(+) cells, were exported from FlowJo. Plots were generated using Seaborn and Matplotlib in Python. Statistical analyses were performed in Python (Scipy and statsmodels). Comparisons of percent GFP(+) cells between strains were analyzed using a linear mixed-effects model with strain, time and media as factors and accounting for repeated measures from the same culture tube. Post-hoc pairwise comparisons between WT and T110A strains within each media-time combination (SPI-2 data: 4 total (2 media, 2 timepoints), SPI-1 data: 6 total (2 media, 3 timepoints)) were performed using unpaired t-tests with p values corrected for multiple comparisons using the Holm-Sidak method. Consistency in trends across replicates was assessed using the sign test. Asterisks denoting significance level are indicated in the Figure legends.

### Fluorescence microscopy

HeLa cells were plated at 6,000 cells/well on fibronectin-coated (10 µg/ml) 96-well glass bottom plates one day prior to infection. Bacterial cultures were prepared as described in the SPI-1 induction assays: Single colonies were inoculated into 3 mL LB-Lennox with appropriate antibiotics and grown shaking at 37°C for 14–16 hours. Overnight cultures were back diluted to an OD_600_ of 0.06 in fresh LB-Lennox and grown shaking for 2.5 hours, then back diluted to an OD_600_ of 0.06 in 5 mL LB-Miller (SPI-1-inducing) or LB-Lennox (control) and grown shaking for 4 hours. One hour prior to infection, HeLa cells were washed three times with imaging media (1x FluoroBrite DMEM, 1% FBS, 2 mM GlutaMAX) to remove residual Penicillin/Streptomycin and 100 µl of imaging media was added to each well. Bacterial cultures were pelleted, resuspended in PBS, and diluted to the indicated multiplicity of infection (MOI). 5 µl of bacteria was added to each well and the plate was incubated at 37˚C for 30 minutes. HeLa cells were washed three times with imaging media to remove extracellular bacteria, and 200 µl of imaging media containing 10 µg/mL gentamicin was added to each well. Plates were sealed with an AeraSeal film and imaged. Imaging was performed with a Nikon Ti2E fluorescence microscope equipped with a Prime BSI sCMOS camera, a SPECTRA III light engine, temperature (37˚C) and environmental (5% CO_2_) control. Images were acquired using a 20x/0.75 numerical aperture objective with 2x2 binning. Acquisition was controlled by Nikon Elements software.

### Image analysis

Nuclei labeled with H2B-miRFP670 were segmented using Cellpose3 and a custom trained model [87,88]. Images with sparse nuclei were poorly segmented due to Cellpose’s standard image normalization and were manually identified and excluded from analysis. All segmented images were manually inspected, and missegmented nuclei were removed. Bacteria labeled with mRuby2 were preprocessed to subtract background and reduce noise using 1) N4 bias field correction algorithm, 2) wavelet-based background subtraction, and 3) anisotropic diffusion [89,90]. Nuclei labels were filtered to remove objects <266 pixels in area (1st percentile of all nuclei). To minimize detection of out-of-focus extracellular bacteria, images were further processed using background subtraction (napari-segment-blobs-and-things-with-membranes: nsbatwm) followed by Laplacian of Gaussian filtering (napari-pyclesperanto-assistant) [91]. Bacteria were segmented using Voronoi-Otsu labeling (pyclesperanto-prototype). A minimum threshold for bacteria object intensity (2500 AU) was determined from uninfected control wells. Bacterial objects were assigned to the nearest nucleus using a Euclidean distance threshold of 60 pixels. Features of nuclei and bacterial objects were extracted using scikit-image regionprops and stored in xarray format. Plots were generated using Seaborn and Matplotlib in Python. Statistical analyses were performed in Python (Scipy and statsmodels) using two-way ANOVA with strain and MOI as factors.

### RNA isolation and quantitative RT-PCR

Following 4 hours of growth in SPI-1-inducing media (LB High Salt), 1 mL of each culture was pelleted (6000 rpm, 2 minutes), snap-frozen and stored at -80˚C. Pellets were brought to room temperature, resuspended in 100 µL TE Buffer with 2 mg/mL lysozyme and incubated for 5 minutes, with vortexing every minute. RNA was isolated using the Monarch Spin RNA Isolation Kit and the Zymo RNA Clean & Concentrator Kit. cDNA was generated using 0.5 µg RNA and the High-Capacity cDNA Reverse Transcription Kit with RNase Inhibitor at 1 U/µL. qRT-PCR was performed on a Quant Studio3 qPCR system by combining 2 µl of diluted cDNA (1:10) with 6 µL of PowerUp SYBR Green Master Mix, 500 nM of each primer (S4.3 Table), and nuclease-free water up to 12 µL. C_T_ values were normalized to the housekeeping gene *ihfB* and the 2^-∆CT^ method was used to calculate relative expression levels. Technical replicates that differed by more than 1.5 C_T_ values were removed from the dataset (1 case across 3 biological replicates, S2 Table). Primer sequences are listed in S4.3 Table.

### Experimental evolution

Glycerol stocks of WT and T110A strains were streaked on LB agar and incubated overnight at 37˚C. Six independent colonies from each strain were inoculated into 3 mL M9/Glc/CAA and grown shaking overnight at 37˚C. The following day, 1 mL of each culture was pelleted (6000rpm, 2 minutes) and washed twice in 1 mL MgM-MES to remove residual M9/Glc/CAA. Washed cells were diluted 1:100 (150 µl in 15 mL MgM-MES in 125 mL flasks) and grown shaking at 37˚C for approximately 24 hours. For 30 days, 150 µl of each culture was transferred daily into 15 mL fresh MgM-MES (1:100) without washing. Glycerol stocks were prepared daily to facilitate subsequent growth assays and whole-genome sequencing.

### Statistical analysis

*Growth curves:* For Fig 1A (i) and S1A pairwise comparisons used two-sided, unpaired t-tests and p values were adjusted for multiple comparisons using the Benjamini-Hochberg FDR method. For Fig 1A (ii) pairwise comparisons used Welch’s t-tests and p values were adjusted for multiple comparisons using the Benjamini-Hochberg FDR method. For S1C Fig (ii) significance was determined using Dunnett’s test. *Flow cytometry:* Comparisons of % GFP(+) cells used a linear mixed-effects model with post-hoc pairwise comparisons performed with unpaired t-tests and Holm-Sidak correction for multiple comparisons. Consistency in trends across replicates was assessed using sign tests. *qRT-PCR:* Significance was determined by Welch’s t-test with Holm-Sidak correction for multiple comparisons. *HeLa cell infection:* Comparisons of infected cell frequency and number of multi-bacteria infection events were performed using two-way ANOVA with strain and MOI as factors. Statistical analyses were performed in Python (Scipy and statsmodels). Alpha set to 0.05. All statistical analyses are reported in S2 Table.

## Supporting information

S1 Fig Growth of WT and T110A strains in multiple media, whole genome sequencing identification of the T110A mutation, and growth curves of the T110A knock-in and revertant strains. (A) Growth curve analysis in additional media conditions. Plate reader growth curves of WT (gray) and MUT (magenta) strains in non-SPI-1-inducing (LB) and SPI-1-inducing (LB-High Salt [LB-HS]) media, replete media (M9/Glc/CAA) and nutrient-limited media (M9/Glc). Growth curves show averages of three independent experiments, each with three technical replicates; shaded regions represent standard deviation. Media control is shown in black. Y-axes are scaled to the growth condition. (ii) Summary growth metrics (lag time, max growth rate) extracted from the data in (i). Individual datapoints represent independent experiments. Asterisks denote significance as determined by two-sided unpaired t-test with Benjamini-Hochberg FDR correction as follows: * p < 0.05, ** p < 0.01; ns, not significant. (B) Whole genome sequencing identified *hns* a328g (T110A) as the only coding region difference between the WT and MUT strains. (C) The *hns* a328g (T110A) mutation was introduced into the WT strain (WT [T110A]) and reverted in the MUT strain (MUT [A110T]). (i) Growth of the knock-in and revertant strains was compared to the original WT and MUT strains in M9/Glc/CAA and MgM-MES. Growth curves show data from a single experiment, with three technical replicates; shaded regions represent standard deviation. Y-axes are scaled to the growth condition. (ii) Summary growth metrics (lag time, max growth rate) for MgM-MES are shown. Individual datapoints represent independent experiments; original and knock-in/revertant strains are shown as filled and unfilled circles, respectively. Significance was determined using Dunnett’s test, comparing the edited strains to the original WT and MUT strains within each media condition (*** p < 0.001; ns, not significant).
(TIF)

S2 Fig Biochemical characterization of H-NS T110A. (A) Schematic of the plasmid construct used for H-NS protein expression. (B) Full SDS-PAGE gel image corresponding to Fig 2B. (C) Sequence and features of the *ssrAB* promoter region used in EMSAs. Underlined sequences indicate PCR primers used to amplify the region from STm genomic DNA. Colored text highlights AT-rich tracts of potential H-NS binding sites (red: longer tracts, blue: shorter tracts). (D) Complete gel images from four independent EMSA experiments for H-NS WT and T110A proteins. Data from replicate 4 is shown in Fig 2C. (E) Quantification and curve fitting of EMSA data from panel D (excluding replicate 4) showing the fraction of DNA-bound H-NS as a function of protein concentration. WT (gray) and T110A (magenta) data points are shown as circles. Lines represent fits to the Hill equation. (F) (i) AlphaFold3 predictions for the interaction of H-NS WT and T110A with the *ssrAB* promoter region across 10 random seeds. The number of predicted hydrogen bonds in the QGR motif is listed for each model. An increased number of hydrogen bonds in the T110A models is consistent with reduced conformational flexibility of the protein, potentially contributing to its reduced DNA-binding affinity. (ii) Probability matrices for the AlphaFold3 model using seed = 2484837.
(TIF)

S3 Fig SPI-2 and SPI-1 reporter expression in H-NS T110A strain. (A) Scatter plots of single-cell sfGFP vs mRuby2 intensity for strains containing the SPI-2 reporter plasmid grown in MgM-MES for 4hrs. Marginal density plots are shown above each axis. sfGFP expression is bimodal, while mRuby2 expression is unimodal and does not correlate with sfGFP in individual cells. Gly = Glycerol, Glu = Glucose. n = 50,000 cells per sample. (B) SPI-2 reporter (P_*ssaG*_-sfGFP(LVA)) expression vs growth. Scatter plot shows % GFP(+) cells versus OD_600_ for WT and T110A samples at 4 hours (corresponding to data in Fig 3B) in MgM-MES with glycerol (circles) or glucose (squares) as the carbon source. (C) Mean fluorescence intensity (MFI) of GFP(+) subpopulations for SPI-2 reporter data shown in Fig 3B. (D) SPI-2 reporter expression under non-inducing conditions. Flow cytometry data were analyzed as in Fig 3B. n = 50,000 cells/sample, 5 independent experiments. (E) Scatter plots of single-cell sfGFP vs mRuby2 intensity for strains containing the SPI-1 reporter plasmid grown in LB High Salt for 4hrs. Marginal density plots are shown above each axis. sfGFP expression is bimodal, while mRuby2 expression is unimodal and does not correlate with sfGFP in individual cells. n = 50,000 cells per sample. (F) MFI of GFP(+) subpopulations for SPI-1 reporter data shown in Fig 3C. (G) SPI-1 reporter (P_*invF*_-sfGFP) expression in the T110A strain in non-inducing media. (i) Representative histogram of WT and T110A strains containing the SPI-1 reporter plasmid grown in LB for 4 hours. n = 50,000 cells. Data corresponds to Fig 3C. (ii) The frequency of SPI-1 reporter GFP(+) cells before induction was assessed by measuring % GFP(+) cells after the first (BD1) and second (BD2) back dilution in LB and comparing to samples grown in SPI-1-inducing media (LB High Salt) for 3, 4, and 5 hours. SPI-1 reporter expression is negligible before induction in LB High Salt. (H) WT, T110A, and WT [T110A] and MUT [A110T] strains containing the SPI-1 reporter plasmid (P_*invF*_-sfGFP) were grown in SPI-1-inducing media (LB High Salt) and non-inducing media (LB) for 4 hrs and analyzed by flow cytometry. (i) Representative histograms at 4 hours for strains grown in LB High Salt. WT and T110A are shown as solid lines, WT [T110A] and MUT [A110T] strains as dashed lines, and the GFP(-) control strain as a black line. (ii) Percentage of GFP(+) cells at 4 hours. Bars represent the mean ± SD of 3 independent experiments. Individual datapoints are shown in shades of gray. n = 50,000 cells per sample. Asterisks denote significance as determined by linear mixed-effects model with post-hoc pairwise comparisons using unpaired t-tests (Welch’s) with Holm-Sidak correction for multiple comparisons: * p < 0.05, ** p < 0.01; ns, not significant. WT strain shown as gray and T110A as magenta across all panels.
(TIF)

S4 Fig Growth, genotype, and SPI-2 reporter expression of evolved clones. (A) Biological replicate of growth curves for WT and T110A evolved clones at d5 and d30 (related to Fig 4A (ii)). (B) Summary of mutations in WT and T110A clones at d5 and d30. Three T110A clones (clones 1, 3, and 4) were sequenced at d5, while all six clones from each background were sequenced at d30. T110A clone 1 d5 had no mutations, but did have some new junction evidence as detailed in S1 Table. (C) (i) Summary of mutations identified in single colonies from selected d30 evolved clones. Colony 0 corresponds to the original sequenced colony (Fig 4B); colonies 1 and 2 are additional single colonies. WT clones are shown on the left (green), T110A clones on the right (blue). Missense mutations, deletions (∆), frameshifts (FS), and nonsense mutations (*) are indicated for each gene. (ii) Growth curves for the additional single colonies (1 and 2) from (i) along with a bulk population sample. Growth metrics (lag time, maximum growth rate, maximum OD_600_) are shown to the right. Filled circles represent the bulk population, open circles the single colonies; WT clones in green, T110A clones in blue; dashed lines indicate the means of the ancestral WT and T110A strains. One experiment shown per clone; ancestral strains included in duplicate; three technical replicates per sample. (D) Loss of SPI-2 induction in evolved clones (related to Fig 4C). (i) Samples were analyzed by flow cytometry after 4 hours in MgM-MES and the percentage of GFP(+) cells is shown. Values represent mean ± standard deviation from four independent experiments. (ii) Mean fluorescence intensity (MFI) of GFP(+) cells. Only T110A clone 2 GFP(+) cells have GFP intensity comparable to the original WT and T110A strains. Other evolved clones show intensities similar to the GFP(-) control, indicating loss of SPI-2 induction. Data shown is mean ± standard deviation of 4 independent experiments. n = 50,000 cells per sample for each replicate.
(TIF)

S1 Table Summary of whole-genome sequencing results corresponding to the data in Figs 1 and 4. (XLSX)

S2 Table Summary of data used for figures and statistical analyses. (XLSX)

S3 Table Reagents. (PDF)

S4 Table Plasmids, Bacterial Strains, and Primers. (PDF)
