# Supplementary material for: Perturbing H-NS function reveals roles in restricting virulence heterogeneity and pathogen adaptation
Source: PLoS Pathog. 2026 Aug 10;22(8):e1014488. doi: 10.1371/journal.ppat.1014488 (PMC13456270; doi:10.1371/journal.ppat.1014488)
Supplement: Supplementary file 1 [file ppat.1014488.s007.pdf]

S3_Table

REAGENT	SOURCE	Catalog #
***Salmonella* Cell Culture**		
LB broth, Miller	Fisher Scientific	BP1426
LB broth, Lennox	Fisher Scientific	BP1427
BactoAgar	Fisher Scientific	DF0140-01-0
BD DIFCO Nutrient Agar	Fisher Scientific	DF0001-17-0
BD Difco Dehydrated Culture Media: M9 Minimal Salts, 5x	Fisher Scientific	DF0485-17
Calcium chloride dihydrate, for molecular biology, ≥99.0%	Sigma-Aldrich	C3306
Magnesium sulfate heptahydrate, BioReagent, for molecular biology, suitable for plant cell culture, ≥99.0%	Sigma-Aldrich	M2773
D-(+)-Glucose,powder, BioReagent, suitable for cell culture, suitable for insect cell culture, suitable for plant cell culture, ≥99.5%	Sigma-Aldrich	G7021
Casamino Acids	Fisher Scientific	BP1424
Potassium chloride, for molecular biology, ≥99.0%	Sigma-Aldrich	P9541
Ammonium sulfate, for molecular biology, ≥99.0%	Sigma-Aldrich	A4418
Potassium sulfate, BioXtra, ≥99.0%	Sigma-Aldrich	P9458
Potassium phosphate monobasic, for molecular biology, ≥98.0%	Sigma-Aldrich	P9791
Mes hydrate,BioPerformance Certified, suitable for cell culture, ≥99.5%	Sigma-Aldrich	M2933
MOPS	Sigma-Aldrich	M3183
Glycerol	Fisher Scientific	BP229-1
Magnesium chloride hexahydrate, BioReagent, suitable for cell culture, suitable for insect cell culture	Sigma-Aldrich	M2393
**Cloning**		
PrimeSTAR Max 2X Premix DNA	Takara Bio	R045B
EcoFlex MoClo Kit	Addgene	Kit # 1000000080
**Mammalian Cell Culture**		
HeLa	ATCC	CCL-2
Lenti-X 293T	Takara	632180
DMEM	Fisher Scientific	MT15017CV
Fetal Bovine Serum	Omega Scientific	FB-02
Penicillin/Streptomycin	Fisher Scientific	15-140-122
GlutaMAX	Fisher Scientific	35-050-061
psPAX2	Addgene	Plasmid #12260
pMD2.g	Addgene	Plasmid #12259
Hygromycin	Invivogen	ant-hg-1

**Plate Reader**		
Glass-bottom 96-well black plate	Tecan	30122306
AeraSeal film	Sigma-Aldrich	A9224
**Whole Genome Sequencing**		
Wizard(R) Genomic DNA Purification Kit	Promega	A1120
**Protein Expression and Purification**		
BL21(DE3) Competent Cells	Thermo Fisher Scientific	EC0114
IPTG (Gold Biotechnology Inc)	Fisher Scientific	50-153-2804
Sodium chloride	Sigma-Aldrich	S5886-1KG
DL-Dithiothreitol,for molecular biology, ≥98% (HPLC), ≥99% (titration)	Sigma-Aldrich	D9779-5G
Phenylmethylsulfonyl Fluoride	Fisher Scientific	52-332-5GM
cOmplete, Mini, EDTA-free Protease Inhibitor Cocktail	Sigma-Aldrich	11836170001
Polyethyleneimine, approx. M.N. 60,000, 50 wt.% aq. solution, branched	Fisher Scientific	AC178571000
1-(3-Aminopropyl)imidazole 97.0+%	Fisher Scientific	A1185100G
2-Mercaptoethanol,for molecular biology, for electrophoresis, suitable for cell culture, BioReagent, 99% (GC/titration)	Sigma-Aldrich	M3148-100ML
Invitrogen Ni-NTA Agarose	Fisher Scientific	R90101
Sodium phosphate dibasic	Sigma-Aldrich	S3264-500G
Corning Cell Culture Phosphate Buffered Saline (1X)	Fisher Scientific	MT21040CV
PreScission Protease	Brickner Lab	
Amicon Ultra Centrifugal Filter, 3 kDa	Sigma-Aldrich	UFC500396
Glycine	Sigma-Aldrich	G8898-500G
Trizma base	Sigma-Aldrich	93362-250G
Sodium dodecyl sulfate	Sigma-Aldrich	L3771-500G
2x Laemmli Sample Buffer	Bio-Rad	1610737
4–15% Mini-PROTEAN TGX Precast Protein Gels, 15-well, 15 µl	Bio-Rad	4561086
Precision Plus Protein Kaleidoscope Prestained Protein Standards	Bio-Rad	1610375
Bio-Safe Coomassie Stain	Bio-Rad	1610786
**EMSAs**		
DNA Clean & Concentrator-5	Zymo Research	D4004
Bromophenol Blue	Fisher Scientific	AAA1846909
Electrophoretic Mobility-Shift Assay (EMSA) Kit, with SYBR Green & SYPRO Ruby EMSA stains	Fisher Scientific	E33075
5% Mini-PROTEAN® TBE Gel, 15 well, 15 µl	Bio-Rad	4565016
Thermo Scientific LightShift Poly (dI-dC)	Fisher Scientific	PI20148E
**Flow Cytometry**		

Electron Microscopy Sciences 16% Paraformaldehyde Aqueous Solution, EM Grade	Fisher Scientific	50-980-487
**Microscopy**		
Fluorobrite DMEM	Fisher Scientific	A1896701
Fibronectin	Sigma-Aldrich	F0895
Cellvis 96 Well glass bottom plate with high performance #1.5 cover glass	Fisher Scientific	NC0536760
Gibco Gentamicin (10 mg/mL)	Fisher Scientific	15-710-064
**RNA isolation and qRT-PCR**		
Lysozyme	Thermo Fisher Scientific	PI90082
Monarch Spin RNA Isolation Kit	NEB	T2110
Zymo RNA Clean & Concentrator Kit	Zymo Research	R1013
High Capacity cDNA Reverse Transcription Kit	Applied Biosystems	4368814
RNase Inhibitor	Applied Biosystems	N8080119
PowerUp SYBR Green Master Mix	Applied Biosystems	A25741
BioDot Pure 96 well plate	DOT Scientific	354-PCR
Plate seal	DOT Scientific	T513
**Software and Algorithms**		
Python3	https://www.python.org/	
Seaborn	https://seaborn.pydata.org	
Matplotlib	https://matplotlib.org/	
Cellpose3	https://github.com/MouseLand/cellpose	
CellTK	https://github.com/braysia/CellTK	
Napari	https://napari.org/stable/	
nsbatwm	https://github.com/haesleinhuepf/napari-segment-blobs-and-things-with-membranes	
clEsperanto	https://github.com/clEsperanto	
Breseq	https://github.com/barricklab/breseq/	
FlowJo	v10.8.2	
Omniplate	https://git.ecdf.ed.ac.uk/pswain/omniplate	
ChimeraX	https://www.rbvi.ucsf.edu/chimerax/	
Fiji	https://imagej.net/software/fiji/	
