# Supplementary material for: Perturbing H-NS function reveals roles in restricting virulence heterogeneity and pathogen adaptation
Source: PLoS Pathog. 2026 Aug 10;22(8):e1014488. doi: 10.1371/journal.ppat.1014488 (PMC13456270; doi:10.1371/journal.ppat.1014488)
Supplement: Supplementary file 2 [file ppat.1014488.s008.pdf]

S4 Table. Plasmids, Bacterial Strains, and Primers

S4.1_Table

Plasmids			
**Plasmid Contents**	**Description**	**Antibiotic Resistance**	**Source**
pTU2-a-PssaG-RiboJ-Bba_B0064-sfGFP-LVA-L3S2P21-SJM901-PET_RBS-mRuby2-Bba_B0012-KanR-pSC101	P_{ssaG}-sfGFP-LVA (SPI-2 Reporter)	Kanamycin	Spratt *et al.* (2025)
pTU2-a-PinvF-RiboJ-Bba_B0064-sfGFP-L3S2P21-SJM901-PET_RBS-mRuby2-Bba_B0012-KanR-pSC101	P_{invF}-sfGFP (SPI-1 Reporter)	Kanamycin	This study
pTU2-a-PROMOTERLESS-sfGFP-L3S2P21-SJM901-PET_RBS-mRuby2-Bba_B0012-KanR-pSC101	Promoterless Control Plasmid	Kanamycin	Spratt *et al.* (2025)
pTU1-B-SJM901-PET_RBS-mRuby2-Bba_B0012-AmpR-pSC101	P_{SJM901}-mRuby2 (Constitutive mRuby2)	Carbenicillin	Spratt *et al.* (2025)
pET-28a-hns-WT-PPX-6xHis	H-NS WT expression plasmid	Kanamycin	This study
pET-28a-hns-T110A-PPX-6xHis	H-NS T110A expression	Kanamycin	This study
pTU1-a-hns_WT_3'end_FRT-chlor-FRT-GalU-p15A	Template for recombineering to revert T110A to WT	Carbenicillin	This study
pTU1-a-hns_T110A_3'end_FRT-chlor-FRT-GalU-p15A	Template for recombineering to introduce T110A to WT	Carbenicillin	This study
pKD46	Arabinose-inducible lambda red proteins, temperature sensitive origin of replication	Carbenicillin	*E. coli* Genetic Stock Center
pCP20	Flpe recombinase, temperature sensitive origin of replication	Carbenicillin	*E. coli* Genetic Stock Center

S4.2_Table

Bacterial Strains		
**Strain contents**	**Description**	**Source**
STm 14028	WT	McClelland lab
STm 14028 *hns* a328g	hns mutant strain	This study
STm 14028 WT P_{ssaG}-sfGFP-LVA	WT with SPI-2 reporter plasmid	Spratt *et al.* (2025)
STm 14028 *hns* a328g P_{ssaG}-sfGFP-LVA	MUT with SPI-2 reporter plasmid	This study
STm 14028 WT P_{invF}-sfGFP	WT with SPI-1 reporter plasmid	This study
STm 14028 *hns* a328g P_{invF}-sfGFP	MUT with SPI-1 reporter plasmid	This study
STm 14028 WT Promoterless Control Plasmid	WT with Promoterless control plasmid	Spratt *et al.* (2025)
STm 14028 *hns* a328g Promoterless Control Plasmid	MUT with Promoterless control plasmid	This study
STm 14028 WT P_{SJM901}-mRuby2	WT with Constitutive mRuby2 plasmid	Spratt *et al.* (2025)
STm 14028 *hns* a328g P_{SJM901}-mRuby2	MUT with Constitutive mRuby2	This study
STm 14028 *hns* a328g (WT [T110A])	a328g introduced to WT	This study
STm 14028 hns g328a (MUT [A110T])	a328g reverted to WT	This study

STm 14028 *hns* a328g (WT [T110A]) P_{invF}-sfGFP	WT [T110A] with SPI-1 reporter plasmid	This study
STm 14028 hns g328a (MUT [A110T]) P_{invF}-sfGFP	MUT [A110T] with SPI-1 reporter plasmid	This study
STm 14028 WT evolved clone 1	WT evolved clone 1 Day 30	This study
STm 14028 WT evolved clone 2	WT evolved clone 2 Day 30	This study
STm 14028 WT evolved clone 3	WT evolved clone 3 Day 30	This study
STm 14028 WT evolved clone 4	WT evolved clone 4 Day 30	This study
STm 14028 WT evolved clone 5	WT evolved clone 5 Day 30	This study
STm 14028 WT evolved clone 6	WT evolved clone 6 Day 30	This study
STm 14028 *hns* a328g evolved clone 1	MUT evolved clone 1 Day 30	This study
STm 14028 *hns* a328g evolved clone 2	MUT evolved clone 2 Day 30	This study
STm 14028 *hns* a328g evolved clone 3	MUT evolved clone 3 Day 30	This study
STm 14028 *hns* a328g evolved clone 4	MUT evolved clone 4 Day 30	This study
STm 14028 *hns* a328g evolved clone 5	MUT evolved clone 5 Day 30	This study
STm 14028 *hns* a328g evolved clone 6	MUT evolved clone 6 Day 30	This study
STm 14028 WT evolved clone 3 P_{ssaG}-sfGFP-LVA	WT evolved clone 3 Day 30 with SPI-2 reporter plasmid	This study
STm 14028 WT evolved clone 5 P_{ssaG}-sfGFP-LVA	WT evolved clone 5 Day 30 with SPI-2 reporter plasmid	This study
STm 14028 *hns* a328g evolved clone 2 P_{ssaG}-sfGFP-LVA	MUT evolved clone 2 Day 30 with SPI-2 reporter plasmid	This study
STm 14028 *hns* a328g evolved clone 5 P_{ssaG}-sfGFP-LVA	MUT evolved clone 5 Day 30 with SPI-2 reporter plasmid	This study

S4.3_Table

**Primers**		
**Purpose**	**Forward (5'-3')**	**Reverse (3'-5')**
Amplify template to introduce a328g (T110A) to WT	ATATAGCTATGTTGACGAAAACGGTGAAACTAAAACCTGG	AAGCGCTGGTAAATCCGGCGCTTTTTTTATGCCTGGGGTC
Amplify template to revert a328g (T110A) to WT	ATATAGCTATGTTGACGAAAACGGTGAAACTAAAACCTGG	AAGCGCTGGTAAATCCGGCGCTTTTTTTATGCCTGGGGTC
invF qRT-PCR	TCCTGAGTTTCGCGCTATTT	GTAACAGCGCCAGTACCTTAT
prgH qRT-PCR	AGGGCCATACATAGTTCGATTAC	CATCACTCTGACCTACCACAAA
ihfB (housekeeping gene) qRT-PCR	TGAAAGACTTGCAACCCAGC	TTTTACCGCGTCTTCAACCG
